# Reviewing inclusivity of the UK bladder and head and neck oncology trial portfolio through eligibility criteria: a scoping review

**DOI:** 10.1186/s13063-026-09424-w

**Published:** 2026-01-08

**Authors:** Georgiana Synesi, Rebecca Lewis, Lucy Kilburn, Judith Bliss, Ka Ching Cheung, Hannah Gribble, Karen Poole, Morgaine Stiles, Emma Hall

**Affiliations:** Clinical Trials and Statistics Unit, The Institute of Cancer Research, London, SM2 5GP UK

**Keywords:** Oncology clinical trials, Eligibility criteria, Equality, Diversity, Inclusivity

## Abstract

**Background:**

In clinical trials, eligibility criteria define a population who may benefit from the intervention without incurring undue safety risks. Despite their value, overly stringent eligibility criteria may pose barriers to inclusive recruitment. They may disproportionately exclude groups of people more likely to be diagnosed at later cancer stages, or have specific co-morbidities which can be associated with socio-demographic or ethnic background. The National Institute for Health and Care Research Innovations in Clinical Trial Design and Delivery for the Under-served (NIHR-INCLUDE) guidance highlighted several groups of people who may be underserved by clinical research. The aim of this review was to assess eligibility criteria in bladder and head and neck oncology trials and consider their potential impact on potentially underserved groups.

**Methods:**

This scoping review included multi-centre, interventional, phase II and III trials in bladder or head and neck cancer, which recruited UK participants between 2013 and 2023. Trials were identified via searches of ClinicalTrials.gov, the International Standard Randomised Controlled Trial Number (ISRCTN) registry, and NIHR Clinical Portfolio Management System (CPMS). Trials’ eligibility criteria were categorised and analysed narratively. Trial parameters (recruitment period, funding type, and whether they were of investigational medicinal products (IMPs)) were cross-tabulated with common exclusion criteria using Fisher’s exact test.

**Results:**

Thirty-eight bladder and 30 head and neck cancer trials met the selection criteria. Twenty-eight out of 68 (41%) had non-commercial sponsors. Fifty-six out of 68 (82%) were of IMPs. Forty-one out of 68 (60%) were industry-funded. Fifty-one out of 68 (75%) were for locally advanced, metastatic, or recurrent disease. Common exclusion criteria relating to pregnancy, performance status, HIV status, and cognitive ability may disproportionately impact some underserved groups suggested by the NIHR-INCLUDE guidance.

**Conclusions:**

This review highlights common exclusion criteria which may disproportionately exclude underserved groups from UK bladder and head and neck oncology trials. To facilitate accessibility to oncology trials, sponsors and triallists should consider how potentially underrepresented groups may be safely included during development of eligibility criteria.

**Supplementary Information:**

The online version contains supplementary material available at 10.1186/s13063-026-09424-w.

## Background

Clinical trial populations should reflect the target population as far as possible, to further understanding of disease mechanisms and ensure generalisability of findings [1]. In interventional clinical trials, eligibility criteria define a trial population who may benefit from a novel treatment whilst protecting their safety [2]. Eligibility criteria often involve characteristics such as disease stage, age, organ function, and concomitant medications [3]. In 1996, S L George categorised justifications for eligibility criteria into scientific (e.g. genetic profile of disease), safety (e.g. excluding patients with poor organ function), and other (e.g. providing informed consent) [4].

Despite their importance, eligibility criteria have often been cited as a barrier to enrolment. Particularly stringent criteria in oncology trials may limit enrolment to patients with a lower healthcare burden [3]. A Canadian study found that a significant proportion of patients who were ineligible for oncology trials may still receive the treatment once it is available in routine practice [5], showing that trial populations may not reflect the heterogeneity of the population who will receive a treatment once it is approved [6]. The stringency of oncology trial criteria may increase the complexity, length, and cost of trials, whilst limiting the generalisability of results to the wider population [4].

The NIHR-INCLUDE guidance highlighted several potentially underserved groups in clinical research, which were categorised into four themes: demographic factors (e.g. people outside of the 18–75 age range), socioeconomic factors (e.g. people who have caring responsibilities or no permanent address), health status (e.g. people who are pregnant or have multiple morbidities), and disease-specific factors (e.g. people with rare diseases) [7]. Eligibility criteria may disproportionately exclude groups of people who are more likely to have certain co-morbid diseases or be diagnosed at certain disease stages. For example, people living in the most socioeconomically deprived areas of England are more likely to be diagnosed with some cancers at an advanced stage compared with those living in the least deprived areas [8].

The American Society of Clinical Oncology (ASCO) and Friends of Oncology issued a joint research statement on the value of broadening eligibility criteria of oncology trials in 2017 [2]. They examined common exclusion criteria (brain metastases, minimum age, HIV status, organ dysfunction, prior/concurrent malignancies) and provided recommendations to expand them with a view to increase inclusivity of oncology trials.

This scoping review focuses on randomised controlled trials (RCTs). RCTs are considered the gold standard for establishing new therapies in oncology clinical trials, with most phase III and an increasing number of phase II trials adopting this design [9, 10]. We chose to investigate trial criteria for bladder and head and neck cancers, as incident populations share some characteristics which are thought to be underrepresented in trials. Both cancers tend to be diagnosed in older people, with the highest incidence rates occurring in those aged 65–69 for head and neck cancers and 85–89 for bladder cancers. They are also diagnosed more frequently in people from disadvantaged socioeconomic backgrounds [11, 12], and there are high reported rates of co-incidence with hypertension [13, 14]. With regard to lifestyle, alcohol and tobacco use are correlated with diagnosis of both cancer types [15]. These characteristics and the ways in which they interact may influence clinical trial eligibility.

The National Cancer Registration and Analysis Service (NCRAS) published data on the disease characteristics of people diagnosed with these cancers in the UK between 2013 and 2022 [16]. During this period, 178,942 people were diagnosed with bladder cancer, of which 96% had cancer developing from urothelial cells. Of these urothelial bladder cancers, 72% were non-muscle-invasive (NMIBC), and 20% were muscle-invasive (MIBC). The majority of NMIBCs (72%) were diagnosed at the earliest stages (Ta/Tis). At diagnosis, the majority of people with MIBC (67%) had localised disease, but 10% of these people were diagnosed with metastatic disease.

Between 2013 and 2022, 101,328 people were diagnosed with head and neck cancers in the UK. Oropharyngeal cancers were the most common, accounting for 34% of diagnoses. Forty-five percent of people with oropharyngeal cancer were diagnosed at the latest stage (stage IV), and between 2019 and 2022, 61% were positive for human papillomavirus (HPV). People with HPV-positive disease have an improved prognosis relative to people with HPV-negative disease [17]. The next most common subsites were the oral cavity and the larynx, accounting for 29% and 18% of diagnoses respectively [16].

During an audit of trials conducted by the Clinical Trials and Statistics Unit at the Institute of Cancer Research (ICR-CTSU) between 2011 and 2021, we reviewed whether eligibility criteria or essential documents were overtly excluding any underserved groups suggested by the NIHR-INCLUDE guidance [18], and confirmed there were no systematic exclusions. The aim of this scoping review was to assess the inclusivity across the UK’s multi-centre bladder and head and neck oncology trials in relation to eligibility criteria and disease characteristics of the incident population, given that they disproportionately impact people from disadvantaged backgrounds.

## Methods

The protocol was devised using the PRISMA extension for scoping reviews checklist [19] and registered prospectively on Open Science Framework [20].

### Eligibility

Included studies were interventional, aimed at assessing the efficacy or safety of treatments for bladder cancer (ICD-10 C67) or head and neck cancers (ICD-10 C00–14, C30–32). They must have been phase II or III, multi-centre, RCTs with at least one site in the UK which recruited patients between 2013 and 2023. Studies were excluded if they were prematurely terminated without recruiting any patients in the specified timeframe, if they had no outcomes relating to patient benefit (e.g. trials solely assessing feasibility or cost efficiency), or if they involved several cohorts of primary tumours which do not fall under C67, C00–14, or C30–32, with the exception of bladder trials where eligibility specified “urothelial carcinoma”.

### Search strategy

A comprehensive search was conducted between February and March 2023 using ClinicalTrials.gov, ISRCTN registry, and NIHR Clinical Portfolio Management System (CPMS), to capture clinical trials of investigational medicinal products (CTIMP) and trials evaluating other (non-IMP) therapies. The search strategy in each database depended on the available information and filtering options. As many filters as possible pertaining to the eligibility criteria were applied in each database. The differences between databases and search criteria are included in Appendix 1.

### Data extraction

Filtered records from all databases were extracted to Microsoft Excel, and duplicates were removed. Initial screening was conducted on the remaining records to exclude inappropriate studies based on their title, start dates, phase, and location. Each record deemed appropriate for full-text screening was independently screened by two reviewers (GS, and either KCC, HG, MS, or SS) with disagreements resolved by a third reviewer (KP). A data charting form was developed by the primary author and refined by the co-authors to determine which variables should be extracted from trial records. Eligibility criteria were organised into four themes for analysis: disease-related factors, co-morbidities, concomitant medications, and patient factors.

### Statistical methods

The specification of co-morbidities and health statuses (pregnancy, HIV status, prior malignancies, central nervous system (CNS) metastases, performance status (PS)) in eligibility criteria was cross-tabulated with trial characteristics (recruitment period, phase, industry funding, whether they were CTIMPs) using Fisher’s exact test. This was to identify trends over time, or any impact of funding source, study design, or intervention relating to these criteria. To assess trends in CNS metastasis criteria, trials in the non-metastatic setting were omitted from the analysis. A *p *value of 0.01 was considered statistically significant to make some account for multiple testing.

## Results

### Systematic search

**Fig. 1:**
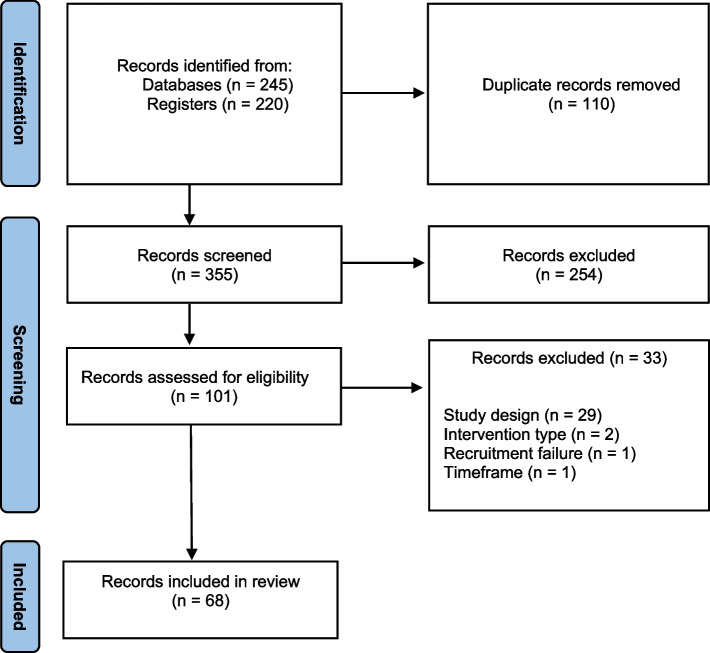
PRISMA flow diagram depicting the selection process

After removing duplicates, the searches retrieved 355 records, of which 254 were excluded after preliminary screening. Full-text screening was carried out for 101 records. Sixty-eight records were selected for inclusion in this review; 38 bladder cancer trial records, and 30 head and neck cancer trial records (Fig. 1). Of the 33 trial records excluded at full-text screening, 29 were excluded due to the trial design. This included trials which did not have a randomised controlled design, trials which were single-centre, and trials which were not phase II or III.

### Trial characteristics

Details about recruitment period, type of funding, phase, type of sponsor, type of trial, and type of intervention are shown in Table 1. The mean number of sites and participants per trial was 111 and 483 for bladder cancer trials, and 83 and 357 for head and neck cancer trials.

**Table 1:** Trial characteristics tabulated by recruitment period and type of funding

	Bladder cancer					Head and neck cancer				
Recruitment period	2013–2017		2018–2023		Total	2013–2017		2018–2023		Total
Type of funding	Non-industry	Industry	Non-industry	Industry		Non-industry	Industry	Non-industry	Industry	
	9	8	1	20	38	9	7	1	13	30
Phase										
II	4	4	0	4	12	1	3	0	7	11
II/III	1	0	0	1	2	0	0	0	2	2
III	4	4	1	15	24	8	4	1	4	17
Trial sponsor										
Non-commercial	9	4	1	4	18	8	0	1	1	10
Commercial	0	4	0	16	20	1	7	0	12	20
Trial type										
CTIMP	3	7	0	20	30	6	7	0	13	26
Non CTIMP	6	1	1	0	8	3	0	1	0	4
Intervention type										
Pharmacological	2	7	0	15	24	2	6	0	11	19
Radiotherapy	2	0	0	0	2	3	0	1	0	4
Surgical	3	0	1	0	4					
Other	2	0	0	0	2					
Mixed regimens	1	0	0	5	6	4	1	0	2	7
Disease stage: bladder cancer										
Any bladder cancer	1	0	1	0	2					
NMIBC	5	1	0	4	10					
MIBC (any)	1	0	0	1	2					
MIBC (non-metastatic)	2	1	0	10	13					
MIBC (metastatic)	0	6	0	5	11					
Disease stage: head and neck cancer										
Non-metastatic or locally advanced						5	1	0	2	8
Metastatic or recurrent						0	6	0	11	17
Multiple disease stages						4	0	1	0	5

Most bladder cancer (*n* = 24/38, 63%) and head and neck cancer trials (*n* = 19/30, 63%) were CTIMPs. These included trials of chemotherapy, immunotherapy, or a combination of the two. Bladder cancer trials categorised as “other” were of hyperthermia and imaging. Trials classed as “mixed regimens” were combinations of chemotherapy, immunotherapy, radiotherapy, and surgery.

### Disease characteristics

Two bladder cancer trials had cohorts including both NMIBC and MIBC. Most trials (*n* = 26/38, 68%) were solely for people with MIBC. Of these, 11/26 were for locally advanced or metastatic disease. Of the 10 trials solely for NMIBC, nine were for intermediate or high-risk disease.

Most head and neck cancer trials were available for people with oropharyngeal (*n* = 28/30, 93%), hypopharyngeal (*n* = 24/30, 80%), laryngeal (*n* = 22/30, 73%), or oral cavity (*n* = 20/30, 67%) cancers. Two trials were available for any anatomical subsite, and one trial was for cancer of the parotid glands only. Twenty-three out of 30 (77%) trials were for metastatic or recurrent disease. Thirteen out of 23 (57%) trials for metastatic or recurrent disease excluded prior therapy in the metastatic or recurrent setting. Ten out of 30 (33%) trials excluded prior treatment with immune checkpoint inhibitors, nine of which were for locally advanced, metastatic, or recurrent disease. Four trials specified inclusion of HPV-positive patients, one specified inclusion of HPV-negative patients, and one trial included separate cohorts for both. Three trials specified inclusion of PD-L1-positive patients.

### Co-morbidities and health status

**Fig. 2:**
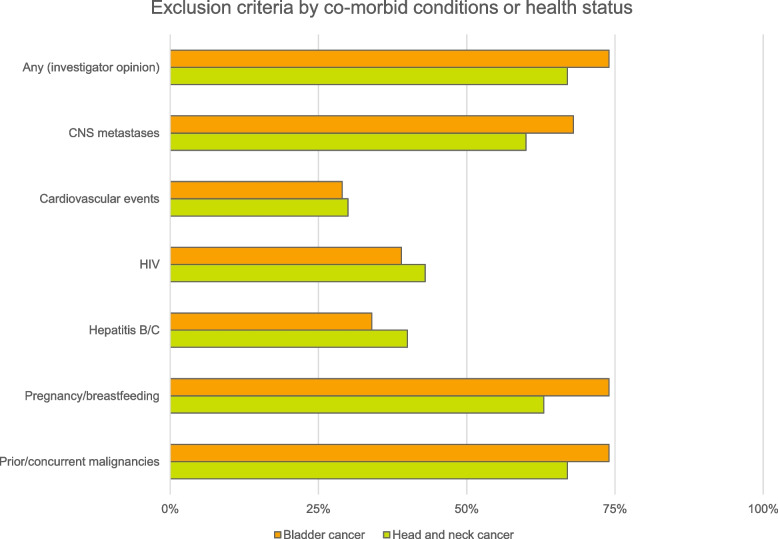
Percentage of trials with exclusion criteria relating to co-morbid conditions and health status. Bladder cancer: *n* = 38 trials. Head and neck cancer: *n* = 30 trials

The most common exclusion criteria pertaining to co-morbidities and health status of all included trials are shown in Fig. 2. Some trials included patients with these conditions if certain circumstances were met (Table 2). Thirty-two out of 48 (47%) trials excluded patients based on the opinion of the local principal investigator. Often, this was justified in terms of safety, or concerns around protocol compliance or lack of informed consent. Eight out of 32 (25%) trials excluded “any other illness” with no further justification. Two out of 32 (6%) trials included phrasing which did not necessarily relate to a medical condition, for example, “Judgement by the Investigator that the patient is unsuitable to participate in the trial”, and “is not in the best interest of the participant to participate, in the opinion of the investigator”. Twenty out of 68 (29%) trials excluded people who had experienced cardiovascular events such as myocardial infarction, stroke, or angina. Twenty-two out of 68 (32%) trials specified adequate renal function, and 20/68 (29%) trials specified adequate hepatic function. Of the trials that did not specify renal or hepatic function, 17 trials listed “adequate organ function” as inclusion criteria.

**Table 2:** Common exclusion criteria and circumstances in which people meeting exclusion criteria would become eligible (if any)

Criteria relating to:	Trials mentioning criteria (*n*, %)
**Prior or concurrent malignancies**	**50/68, 74%**
Exclude all	9/50, 18%
Include if disease-free 2 years	9/50, 18%
Include if disease-free 3 years	16/50, 32%
Include if disease-free 5 years	16/50, 32%
**Pregnancy or breastfeeding**	47/68, 69%
Exclude all	47/47, 100%
**People of childbearing potential**	47/68, 69%
Exclude all	0/47, 0%
Include if not pregnant or breastfeeding	10/47, 21%
Include if effective contraception used	37/47, 79%
**CNS metastases**	44/68, 65%
Exclude all metastases	18/44, 41%
Exclude all CNS metastases	4/44, 9%
Include if treated/asymptomatic	22/44, 50%
**HIV**	28/68, 41%
Exclude all	21/28, 75%
Include if undetectable viral load	7/28, 25%
**Hepatitis B or C**	25/68, 37%
Exclude all	8/25, 32%
Include if undetectable viral load	17/25, 68%

Thirty-eight out of 68 (56%) trials excluded patients with a WHO/ECOG PS score of >1. This was particularly apparent for head and neck cancer trials: only 2/30 (7%) trials included patients with a maximum score of 2, whereas 17/38 (45%) of bladder cancer trials included patients with a score of ≥2. Twenty-eight out of 38 (74%) trials excluding patients with a PS score of >1 were of locally advanced, metastatic, or recurrent disease. Forty-seven out of 68 (69%) trials did not specify a minimum life expectancy. Of those that did, 17/21 (81%) had outcome measures to be assessed within the minimum life expectancy period. Twelve out of 21 (57%) were for locally advanced or metastatic disease (six bladder, six head and neck), and 3/21 (14%) were for intermediate or high-risk disease. Most required 10–12 weeks’ life expectancy (18/21, 86%), but 3/21 (14%) required 6 months. Six out of 13 (46%) bladder trials specifying a minimum life expectancy were for non-metastatic disease, compared with only 1/8 (13%) head and neck trials. Six out of 13 (46%) bladder cancer trials stipulating a maximum PS score of 2 also had minimum life expectancy requirements of between 3 and 6 months. Of the four trials which included patients with a maximum score of 3–4, one had a life expectancy requirement of 6 months. Seven out of 25 (28%) head and neck cancer trials stipulating a maximum PS score of 1 also had life expectancy requirements of 3–6 months.

**Fig. 3:**
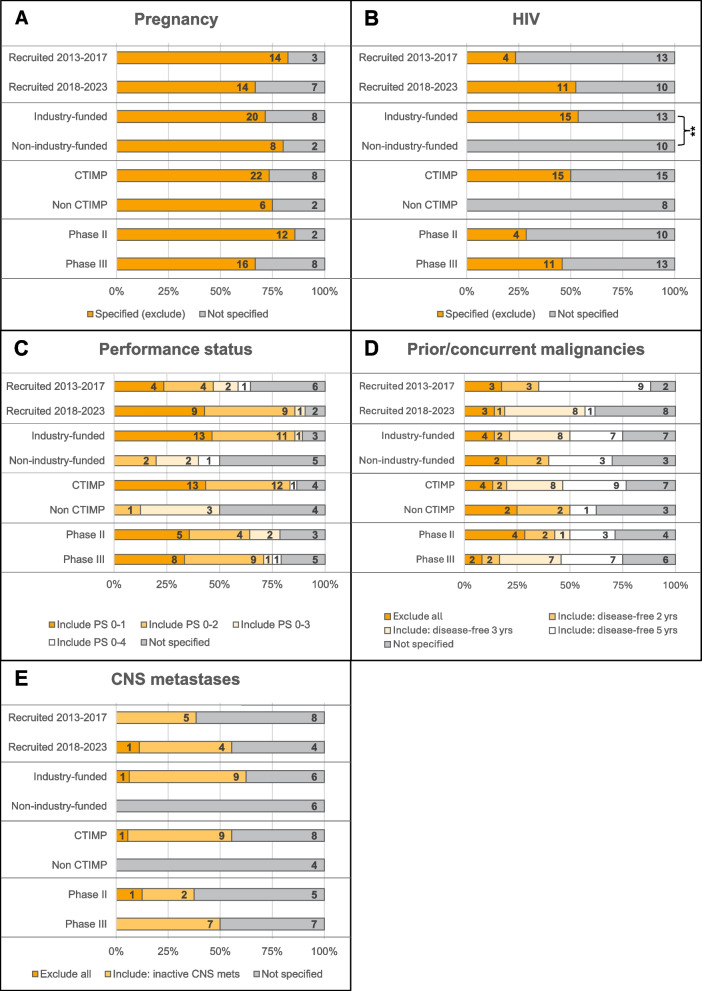
Bladder cancer trial characteristics cross-tabulated with health status eligibility criteria (**p < 0.01, Fisher’s exact test). **A**–**D** *n* = 38 trials. **E** *n* = 22 trials (trials in non-metastatic setting excluded from analysis)

**Fig. 4:**
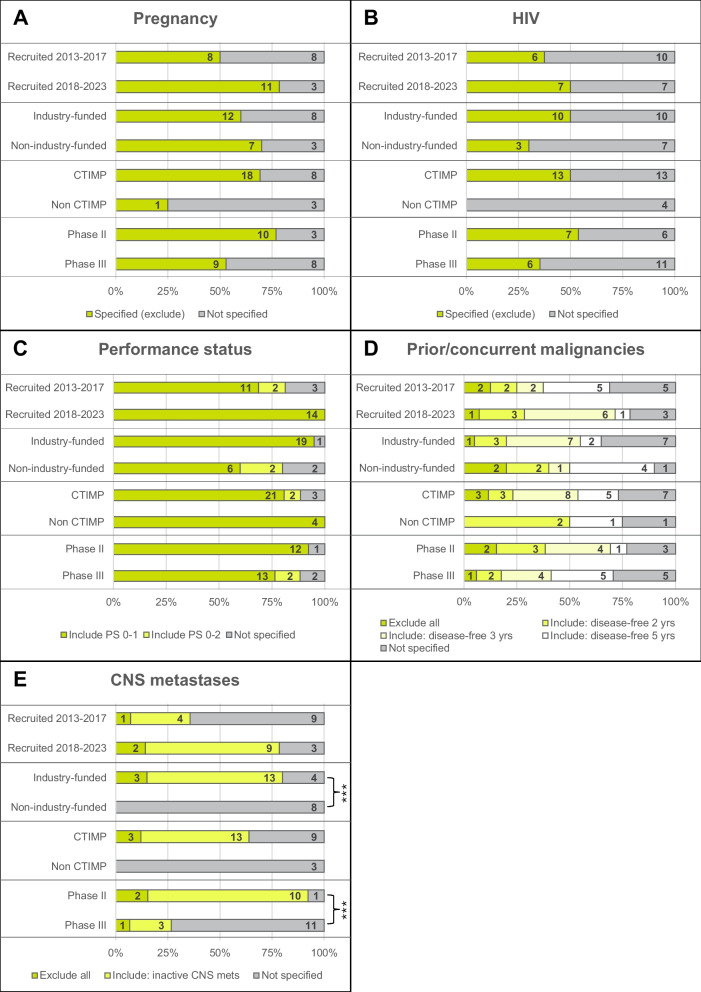
Head and neck cancer trial characteristics cross-tabulated with health status eligibility criteria (***p < 0.001, Fisher’s exact test). **A**–**D** *n* = 30 trials. **E** *n* = 28 trials (trials in non-metastatic setting excluded from analysis)

Although not statistically significant, Fig. 3B shows some patterns regarding HIV criteria and recruitment period; 4/17 (24%) studies recruiting between 2013 and 2017 specified HIV criteria, a percentage which more than doubled amongst studies recruiting between 2018 and 2023 (11/21, 52%). Furthermore, all trials excluding people with HIV were of IMPs, with 14/15 (93%) including an immunotherapy component. Including patients with a positive HIV status was found to be significantly different according to funding (*p* = 0.003) in bladder cancer trials (Fig. 3B), with only industry-funded trials excluding people with HIV. Amongst head and neck cancer trials, criteria relating to CNS metastases were specified more frequently amongst industry-funded (*p* < 0.001) and phase II (*p* = 0.001) trials compared with non-industry-funded and phase III trials respectively (Fig. 4E). No other statistically significant differences were observed (Figs. 3 and 4).

### Concomitant medication

Immunomodulators (including live attenuated vaccines) were the most commonly excluded concomitant medication (25/68 trials, 37%). Regarding concurrent anti-cancer therapy, the most commonly excluded medication was another IMP during the course of the trial (20/68, 29% - all CTIMPs). Two trials excluded patients undergoing any concurrent anti-cancer treatment, both of which were CTIMPs.

### Patient factors

Sixty-seven out of 68 (99%) trials stipulated criteria around informed consent. Nine out of 68 (13%) trials stated exclusion criteria for those without capacity to provide informed consent. Zero out of 9 trials allowed consent to be given on their behalf. Conversely, 3/68 (4%) trials stipulated that a legal representative could consent on behalf of participants without capacity to do so. The remaining trials made no reference to capacity or ability to consent.

Thirty-eight out of 68 trials (56%) specified age limits. Thirty-two out of 38 (84%) of these had a lower age limit of 18 years old, but six trials included participants as young as 16. Two trials specified an upper age limit of 70 years old. Both were CTIMPs, for head and neck cancer.

### Impact on underserved groups

Table 3 maps eligibility criteria to underserved groups from the NIHR-INCLUDE guidance [7] to assess the impact of common criteria on the recruitment of participants belonging to underrepresented groups.

**Table 3:** The potential impact of eligibility criteria on the recruitment of underserved groups, according to NIHR-INCLUDE guidance

Underrepresented groups	Relevant criteria identified	Quotes from inclusion/exclusion criteria
**Demographic factors**		
Age extremes (e.g. under 18 and over 75)	• 6/68 trials included participants under 18 • 2/68 trials excluded participants over 70	
**Social and economic factors**		
People living in remote areas	• One trial noted geographical location as a potential exclusion criterion	• “Any condition (e.g. psychological, **geographical**, etc.) that does not permit compliance with the protocol”
People not fluent in the majority language	• Two trials discussed language as a potential barrier to completing quality of life questionnaires o One trial stipulated fluency for inclusion, and one did not	• “Patient is able (**i.e. sufficiently fluent**) and willing to complete the quality of life questionnaires” • “Has completed the health-related quality of life questionnaires or is unable to complete them because of literacy, **insufficient English** or limited vision”
**Health status**		
Mental health conditions	• 10/68 trials explicitly exclude patients with psychiatric, social, or addiction disorders • 32/68 trials exclude patients with any condition the principal investigator deems incompatible with participation	• “Has a known psychiatric or substance abuse disorder” • “Any serious and/or unstable pre-existing medical (aside from malignancy), psychiatric disorder, or other condition that could interfere with participant’s safety, obtaining informed consent, or compliance to the study procedures in the opinion of the investigator”
People who lack capacity to consent for themselves, or those with cognitive impairment	• 9/68 trials stated exclusion criteria for patients unable to provide informed consent • 3/68 trials allowed consent to be given by a caregiver or legal representative for those without capacity • 4/68 trials excluded patients without the cognitive ability to complete quality of life questionnaires • 4/68 trials using quality of life questionnaires did not stipulate cognitive ability as an inclusion criterion	• “Able to give informed consent, indicating the patient has been informed of and understands the experimental nature of the study” • “Must sign an informed consent form (or their legally acceptable representative must sign)” • “The capacity to understand the patient information sheet” • “Adequate cognitive ability to complete quality of life assessments” “Any condition (e.g. **psychological**, geographical, etc.) that does not permit compliance with the protocol”
Pregnant women	• 47/68 trials excluded people who are pregnant or lactating	• “Female participants: must not be pregnant, not breastfeeding, and… agree to use a method of birth control from 30 days prior to randomization and for at least 120 days after the last dose of study treatment”
Too severely ill	• 53/68 trials excluded patients with a PS of >2	
**Disease-specific factors**		
Rare diseases and genetic disease subtypes	• 11/68 trials included patients based on their HPV or PD-L1 status, or presence of FGFR alterations	
People in cancer trials with brain metastases	• 28/68 trials were in the metastatic setting o 21/28 excluded patients with active CNS metastases o 3/28 excluded patients with inactive CNS metastases o 4/28 did not specify that this group should be excluded	

## Discussion

The findings of this review suggested that common bladder and head and neck cancer trial eligibility criteria may disproportionately exclude some underrepresented groups identified by the NIHR-INCLUDE guidance. We also identified some differences in practice by funding type and phase.

The majority of bladder cancers are diagnosed at an early stage in the UK, and the majority of head and neck cancers are diagnosed at a more advanced stage [16]. We identified a focus on advanced or metastatic disease across both cancer types. These data may suggest that people with less advanced disease may have limited access to bladder cancer trials. Many trials in advanced disease settings were industry-funded and sponsored CTIMPs, which likely reflects the practice of testing efficacy of novel therapies in patients whose disease has progressed following standard treatment [21]. Most head and neck trials were available for oropharyngeal cancers, reflecting the majority of diagnoses in the incident population. All trials specifying participant HPV status (positive or negative) were available for people with oropharyngeal cancer, which is often associated with HPV-positive status [16]. The risk of developing laryngeal cancer is 5.4× higher amongst people with HPV-positive status [22], but only 2/6 trials specifying HPV status were available to those with laryngeal cancer. Only 2/6 trials specifying HPV status were available for HPV-negative participants, which is more common amongst people who are socioeconomically disadvantaged [23]. People identifying as transgender may be less likely to have been vaccinated against HPV and at increased risk of becoming infected [24]. HPV-related criteria may therefore alter accessibility of head and neck cancer trials for socioeconomically disadvantaged or transgender patients.

In 2017, there were an estimated 101,600 people living with HIV in the UK, including 4370 new diagnoses. Fifty-three percent of new diagnoses were in gay and bisexual people, compared to 18% heterosexual men and 24% heterosexual women. Individuals from Black African ethnic groups comprised 38% heterosexual adults with new diagnoses [25]. UK primary care data showed that transgender people are more likely to have HIV than cisgender people [26]. Given that HIV disproportionately affects these groups, routine exclusion of people living with HIV may contribute to their underrepresentation in bladder and head and neck cancer trials. A much higher proportion of bladder trials recruiting from 2018 to 2023 specified HIV exclusion criteria relative to earlier trials, and all of these were CTIMPs. This finding may be explained by the movement towards immunotherapy oncology treatments, and every bladder trial recruiting from 2018 onwards in this review involved an immunotherapy component. This coupled with the fact that industry-funded bladder cancer trials were significantly more likely to exclude people living with HIV may reflect safety concerns around interactions with novel agents being tested and HIV therapies, and around interpretation of trial results. Reflecting this, a review of 297 US CTIMP oncology trial protocols revealed only five included HIV-positive participants [3]. It has been recommended to include people living with HIV with a low risk of developing AIDS unless there is specific rationale to exclude them. Receiving antiretroviral therapy should not be exclusionary unless it is known to interact with the trial treatment [2]. Ten out of 28 (36%) trials included people with a history of HIV in line with these recommendations, suggesting that there is scope to increase accessibility to bladder and head and neck cancer trials for people living with HIV by adhering to these recommendations.

Our findings suggest that there are limited opportunities for pregnant people to join clinical trials for bladder or head and neck cancers. This is likely due to the advanced age of the incident population (meaning pregnancy is unlikely) and concerns around foetal exposure to treatment. Irradiation to the head and neck exposes the foetus to radiation even when abdominal and pelvic shields are used [27]. This may result in structural malformation, organ dysfunction, growth retardation, teratogenesis, and foetal death [28]. Ten out of 68 (15%) trials did not explicitly exclude pregnant participants, eight of which involved immune checkpoint inhibitors. A US study of around 2500 reports of cancer treatment with immune checkpoint inhibitors during pregnancy found that their use was not associated with maternal, foetal, or post-natal adverse effects when compared with other anti-cancer treatments [29]. These findings suggest that oncology trials investigating immune checkpoint inhibitors may be able to safely include pregnant people, and protocol development personnel should consider this when designing eligibility criteria.

Compared with people from white ethnic backgrounds, people from Asian ethnic backgrounds are more likely to have cardiovascular disease, and people from Black ethnic backgrounds are more likely to have hypertension or a stroke [30]. All 20 trials with cardiovascular exclusion criteria were CTIMPs of chemotherapy or immunotherapy. The summary of product characteristics for 18/20 agents in these trials stated potential cardiovascular adverse effects, suggesting that although trials with these criteria may disproportionately exclude people from Black and Asian ethnic backgrounds, this is likely due to safety concerns with the agents being tested.

We found that people with CNS metastases were routinely excluded from bladder and head and neck oncology trials. Exclusions relating to CNS metastases being more likely in industry-funded and phase II trials are likely to reflect concerns around IMPs and assessing their safety and efficacy in earlier phase trials. However, 22/26 (85%) trials which specified criteria around CNS metastases included participants with inactive metastases, in line with recommendations [2]. No trial protocols permitted the inclusion of participants with symptomatic metastases. Similarly, people with prior or concurrent malignancies should be included where risk of interference with the trial treatment and assessment of endpoints is low [2]. Biological males and people with a history of smoking or alcoholism may be at higher risk of developing multiple primary cancers [31]. Higher rates of smoking were observed amongst those without any formal qualifications and those who were unemployed, according to the England and Wales Census 2021 [32]. These relationships exemplify how exclusion criteria may bias against inclusion of individuals at socioeconomic disadvantage.

All trials which explicitly excluded patients without capacity to consent were of advanced, high-risk, or recurrent disease. A metastatic cancer study found that 54% of participants with non-CNS metastases showed impaired capacity to consent [33]. Due to ethical concerns about vulnerability, people with impaired capacity to consent are underrepresented in several areas of clinical research [34]. To mitigate this, the NIHR-INCLUDE Impaired Capacity to Consent Framework [35] recommends that legal representatives should be approached for consent where possible. Given the small number of trials included in this review which allowed third party consent, protocol development personnel should consider whether future trials could feasibly implement this to improve accessibility to bladder and head and neck cancer trials for people with impaired capacity.

The incident populations of bladder and head and neck cancers tend to be older adults, with peak incidence in patients aged 85–89 and 65–69 respectively [11, 12]. A review of 742 oncology RCT protocols showed that only 10% imposed an upper age limit [36]. This coupled with the small number of trials within this review with an upper age limit suggests that older adults are not often directly excluded from trial participation based on their age. However, given that over 50% of adults aged 65 and older have at least two co-morbidities [37], this group may be disproportionately excluded by other criteria. Conversely, whilst people aged under 18 are typically excluded from participating in CTIMP drug trials, it has been recommended that sponsors should consider enrolling 12–17 year olds onto adult oncology trials where tumour biology or the mechanism of action of the drug is equivalent to adults [38]. However, due to the higher age of incidence observed in bladder cancer, the exclusion of paediatric patients from these oncology trials is not likely to significantly impact their inclusivity.

More than 80% of trials with minimum life expectancy requirements had outcome measures to be assessed within that period, which may reflect sponsors wanting to recruit patients who are less severely ill for both safety and follow-up purposes. In elderly patients with bladder cancer, patients with Karnofsky PS score of 80 or below (equivalent to WHO/ECOG 2+) had a 1.8× greater risk of death than those with a score above 90 (equivalent to WHO/ECOG 0–0.5) [39]. However, given that the majority of trials stipulating a maximum PS score of 1 were for people with locally advanced, metastatic, or recurrent disease, excluding patients who are severely ill may limit the generalisability of findings from these trials. Over 80% of trials with a maximum PS score of 1 were of targeted therapies. In a questionnaire to attendees of an ASCO-American Association of Cancer Research Methods in Clinical Cancer Research workshop, mostly comprising physicians, 53% of respondents felt that trials of targeted therapies should include patients with an ECOG PS of 2 [40]. These findings suggest that protocol development personnel, particularly in targeted therapy trials, should consider whether PS criteria could be broadened to include participants who better reflect the population who will receive the treatment once widely available.

### Limitations and recommendations

This scoping review was based on publicly available records, so we did not have access to whole trial protocols. Reviewing trial records by this method relies on them being kept up to date by trial teams, and therefore potential data inaccuracies may be a limitation. This review relates to two disease subtypes based on clinical trials conducted in the UK. Further work across disease subtypes and countries of conduct is needed to build evidence on the impact of eligibility criteria on inclusivity in oncology trials.

In the UK, there remains a lack of specific guidance to encourage triallists and sponsors to consider how they can design inclusive trial criteria, which may in part explain the findings of this review. Two major regulatory bodies, the Health Research Authority and the Medicines and Healthcare products Regulatory Agency are currently piloting inclusion and diversity guidance. This guidance encourages triallists to rationalise the specification of eligibility criteria which may influence the participation of underserved groups, but it is not a mandatory requirement [41]. We hope that the findings of this scoping review will encourage sponsors and triallists in this and other oncology settings to consider inclusivity when designing eligibility criteria. Whilst this review has revealed common exclusion criteria, it highlights that their implementation should be considered on a trial-by-trial basis. In cases where there are concerns about including patients with certain conditions, triallists could consider including specific cohorts to facilitate equitable access to clinical trials. Additionally, the target population for whom the intervention is intended to be used should be considered; for example, if the intervention will be available to patients regardless of life expectancy and performance status, triallists should consider whether related exclusion criteria are appropriate. Albeit important, we acknowledge that reassessing trial eligibility criteria is just one aspect of improving access to and inclusivity of clinical trials. For sponsors and triallists striving to conduct inclusive non-commercial bladder and head and neck oncology trials, we recommend checking inclusivity of trials through robust collection and monitoring of demographic data from trial participants [42] against characteristics of incident populations, and regularly reviewing practice to identify any areas for improvement [18]. Specifically, sponsors and triallists should consider how potentially underrepresented groups may be safely included in clinical research during the trial design stage.

## Conclusions

This review indicates common exclusion criteria which may be disproportionately excluding underrepresented groups from bladder and head and neck oncology trials in the UK. Particularly, during protocol development, it should be carefully considered whether it is necessary to impose criteria which exclude all people living with certain co-morbid conditions without exception. Although eligibility criteria are necessary to define the trial population, the findings of this review in the context of published literature suggest that there may be scope to broaden criteria to include a wider range of participants where it is safe to do so.

## Supplementary Information

Supplementary Material 1.

Supplementary Material 2.

## Data Availability

The datasets used and/or analysed during the current study are available from the corresponding author on reasonable request.

## Abbreviations

ASCO: American Society of Clinical Oncology
CNS: Central nervous system
CPMS: Clinical Portfolio Management System
ECOG: Eastern Cooperative Oncology Group
HIV: Human immunodeficiency virus
HPV: Human papillomavirus
ICR-CTSU: Clinical Trials and Statistics Unit at The Institute of Cancer Research
IMP: Investigational medicinal product
INCLUDE: Innovations in Clinical Trial Design and Delivery for the Under-served
ISRCTN: International Standard Randomised Controlled Trial Number
MIBC: Muscle-invasive bladder cancer
NCRAS: National Cancer Registration and Analysis Service
NIHR: National Institute for Health and Care Research
NMIBC: Non-muscle-invasive bladder cancer
PS: Performance status
RCT: Randomised controlled trial
WHO: World Health Organization
