# Supplementary material for: Reviewing inclusivity of the UK bladder and head and neck oncology trial portfolio through eligibility criteria: a scoping review
Source: Trials. 2026 Jan 8;27:19. doi: 10.1186/s13063-026-09424-w (PMC12790708; doi:10.1186/s13063-026-09424-w)
Supplement: Supplementary file 1 — Supplementary Material 1. [file 13063_2026_9424_MOESM1_ESM.pdf]

Appendix 1: Databases and search criteria

Database/registry	Type of record	Type of research	Filter categories applied	Search terms
ClinicalTrials.gov (US)	Clinical trials	• Interventional/ observational<br>• CTIMPs and medical devices	• Cancer type<br>• Location<br>• Phase<br>• Recruitment dates<br>• Study design	• “Bladder cancer”<br>• “Urothelial cancer”<br>• “Head and neck cancer”<br>• “HNSCC”
ISRCTN Registry (UK)	Clinical trials	• Interventional/ observational<br>• CTIMPs and medical devices	• Cancer type<br>• Phase	• “Bladder cancer”<br>• “Urothelial”<br>• “Head and neck cancer”<br>• “Larynx”<br>• “Pharynx”<br>• “Oral cancer”
NIHR CRN Portfolio (UK)	Clinical trials and research studies	• Interventional/ observational<br>• Non-commercial funding	• Cancer type<br>• Geographical scope<br>• Phase<br>• Study design	• “Bladder cancer”<br>• “Head and neck cancer”<br>• “Head and neck/Oncology”

Appendix 1 shows the differences between databases and registries used in this scoping review, along with the search terms used in each.
